# An Algorithm That Predicts CSI to Allocate Bandwidth for Healthcare Monitoring in Hospital's Waiting Rooms

**DOI:** 10.1155/2012/843527

**Published:** 2012-03-22

**Authors:** Di Lin, Fabrice Labeau

**Affiliations:** Department of Electrical and Computer Engineering, McGill University, Montreal, QC, Canada H3A 2A7

## Abstract

In wireless healthcare monitoring systems, bandwidth allocation is an efficient solution to the problem of scarce wireless bandwidth for the monitoring of patients. However, when the central unit cannot access the exact channel state information (CSI), the efficiency of bandwidth allocation decreases, and the system performance also decreases. In this paper, we propose an algorithm to reduce the negative effects of imperfect CSI on system performance. In this algorithm, the central unit can predict the current CSI by previous CSI when the current CSI is not available. We analyze the reliability of the proposed algorithm by deducing the standard error of estimated CSI with this algorithm. In addition, we analyze the efficiency of the proposed algorithm by discussing the system performance with this algorithm.

## 1. Introduction

**Figure 1:**
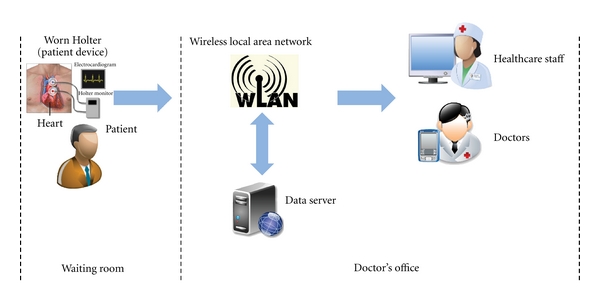
Architecture of our healthcare monitoring system.

 The increasing number of cases on waiting room death, which refers to the death of patients while staying in a hospital's waiting room to be given a medical examination, underscores the significance of improving healthcare quality [1]. Most of these cases occur when patients are left alone in waiting rooms, such as when healthcare staff are taking a break or being busy performing other clinical and non-clinical functions. As a potential way of improving healthcare quality, a wireless healthcare monitoring system (illustrated in Figure 1 and detailed later) could help healthcare staff monitor the condition of patients by automatically collecting patient's data, making some initial decisions on patient condition, and transmitting these decisions and medical data to a doctor's office via wireless local area network (WLAN). Once emergent condition of a particular patient occurs, healthcare staff would be alerted.

From a network design perspective, a wireless healthcare monitoring system should be capable of supporting the number of patients that will be using the system; being able to assess the network's capability to serve a given number of patients is a critical factor in promoting adoption of such systems. Therefore, the *network patient capacity*, which we define as the number of patients that one WLAN deployment can support, is a critical design criterion and performance metric for wireless healthcare monitoring systems. From a practical standpoint, if the hospital's patient capacity exceeds the network patient capacity, then another WLAN will need to be deployed in parallel within the hospital. Beyond the cost of deploying several networks in parallel, their co-existence might also reduce their individual performance. As an example, it is advisable to separate different access points by at least 100 meters (at a transmit power of 500 mW [2]), in order to avoid cochannel interference; this constraint would not be realistic in most hospital environments, so that decreased network performance would be unavoidable. These considerations lead us, in this paper, to attempt to maximize the network patient capacity of a given WLAN deployment, considering real-world transmission scenarios and quality-of-service (QoS) requirements. Through reasonable bandwidth allocation, network patient capacity can be maximized if the central unit for bandwidth allocation can access perfect channel state information (CSI). However, in reality, CSI is always imperfect due to channel estimation errors, feedback errors, and feedback failure. These types of imperfect CSI would decrease the efficiency of bandwidth allocation.

This paper contributes to a channel prediction-based algorithm for bandwidth allocation within an in-hospital monitoring system, in consideration of both the effect of using imperfect previous CSI to predict current CSI and the channel models applicable for hospital environments. To show the reliability of our proposed scheme, we compare the standard error of our proposed algorithm and that of the algorithm without channel prediction, which is viewed as a benchmark (the algorithms proposed in [3–6] cannot be used as benchmarks since they have the assumption of perfect previous CSI for channel prediction, and this assumption does not hold in reality). Then, to show the efficiency of our proposed scheme, we compare the system performance with our proposed scheme and that without channel prediction.

The paper is organized in the following. In Section 2, we introduce the background context as well as the related work on channel state prediction. Then, in Section 3, we discuss the scheme of bandwidth allocation in the cases of perfect CSI and of imperfect CSI. In Section 4, we clarify our channel prediction-based algorithm to reduce the negative effects of imperfect CSI. In Section 5, we analyze the standard error of our proposed algorithm. In Section 6, we discuss the simulation results. In Section 7, we conclude this paper.

## 2. Background Context and Related Work

 In this section, we first clarify the architecture of our monitoring system by taking monitoring patients with heart diseases as an example. Next, as the background for our study, we introduce several possible types of imperfect CSI and channel models in the WLAN for healthcare monitoring. Finally, we present the related work on the prediction of channel state information.

### 2.1. Architecture of Our Health Monitoring System

 Based on their coverage, healthcare monitoring systems can mainly be classified into two types: in-hospital monitoring systems [7–9] and remote monitoring systems [10–12]. In-hospital monitoring systems serve inpatients who usually require intensive watching. Once abnormal conditions occur, the healthcare staff must be alerted in time since a delay of even a few seconds may sometimes mean a loss of life. Remote monitoring systems mainly serve the elderly or chronic patients, and emergent cases do not occur frequently.

Cypher et al. introduce a method to select an appropriate network for in-hospital healthcare applications from current available network candidates [13]. The selection process considers several factors, including the required bandwidth, the coverage area, and the network architecture. It is concluded in [13] that a BAN is the most appropriate for data transmission from sensors to patient computing devices, and a WLAN should be used for data transmission from patient computing devices to the doctor's office.

In this paper, we focus on the monitoring of patients at the waiting room, so it is an in-hospital monitoring system. To clarify the architecture of an in-hospital monitoring system, we take the monitoring of patients with heart diseases as an example (the architecture of monitoring heart diseases is shown in Figure 1). Once arriving, a patient would be required to wear a Holter device at his waist, shoulder, or neck. The Holter device would collect the electrocardiogram (ECG) of this patient and regularly send the ECG to doctors for diagnosis as well as to a data server for filing (the information about ECG is listed in Table 1). Within this monitoring system, we use the IEEE 802.11n based WLAN, the most recently published IEEE standard for WLAN, for data transmission and communication. IEEE 802.11n employs both the technologies of MIMO and OFDM, so the amount of bandwidth in WLAN equals the number of subcarriers. We would interchangeably use these two terms *bandwidth* and *subcarrier* in the following.

As shown in Figure 1, a WLAN is responsible for the transmission of traffic between patient devices and the doctor's office. Due to the limited memory size of patient computing devices, medical data should be transmitted from patient devices to the doctor's office; building on the status of patients, the data from different patient devices may have different delay requirements and should be given different priorities. In addition, patients and healthcare staff may communicate via video conferences when abnormal status is detected [14]. Finally, some messages on patient information also need to be sent between patient devices and a computer or data server in the doctor's office. Therefore, the traffic in the WLAN mainly include three categories: messages, video conferences, and medical data with different priorities.

### 2.2. Types of Imperfect CSI

**Figure 2:**
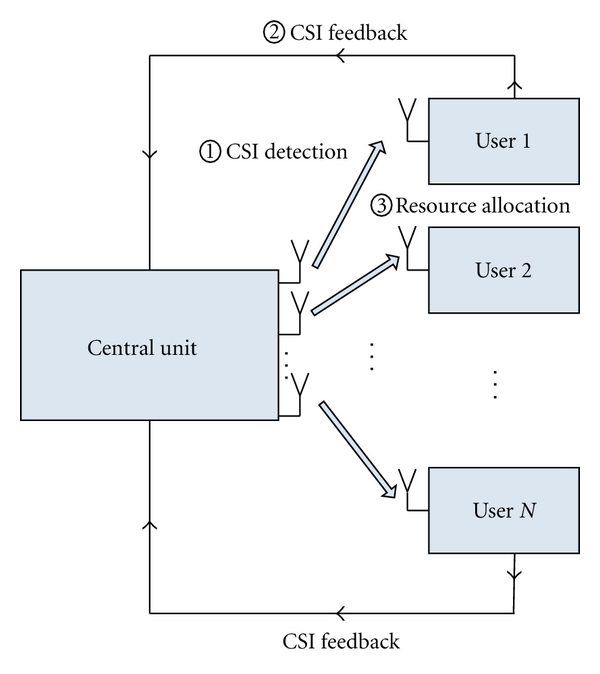
Information flow of a centralized network [15].

 In a healthcare monitoring system, the information flow for bandwidth allocation in the WLAN is illustrated in Figure 2 [15]. Figure 2 shows that three steps are required for bandwidth allocation in one time slot. The first step is to detect the conditions of various channels. Specifically, the central unit sends pilot signals, the amplitudes of which are known by all users. Then, each user estimates the channel fading by comparing the amplitudes of received signals and those of transmitted signals. The second step is CSI feedback; that is, each user sends the estimated CSI to the central unit for bandwidth allocation. In the third step, the central unit allocates wireless bandwidth among users based on the CSI feedback and, then, sends the decision of bandwidth allocation to each user.

Building on the information flow shown in Figure 2, there are mainly three types of potential causes of imperfect CSI in the context of healthcare monitoring. We discuss these types of imperfect CSI and their respective causes in the following.

The first type of imperfect CSI is caused by the errors of forward channel detection in step one, shown in Figure 2 [16], and we name it forward-channel detection-based imperfect CSI (ForCD-ICSI). In a noisy forward channel, a difference exists between the detected channel fading and the exact channel fading. Therefore, the central unit cannot send an exact CSI to users.

The second type of imperfect CSI is caused by the errors of CSI feedback in step two, shown in Figure 2 [5], and we name it feedback-based imperfect CSI (Fe-ICSI). In a noisy feedback channel, a difference exists between the detected channel fading and the exact channel fading. Therefore, the central unit cannot receive the exact CSI feedback from some users. If we employ the automatic repeat request (ARQ) in the upper layers, the channel detection errors would lead to a feedback delay. If the feedback delay is larger than a time slot for bandwidth allocation, the central unit cannot obtain the detected CSI in the current time slot, which would reduce the efficiency of bandwidth allocation.

The third type of imperfect CSI is caused by the compression of feedback CSI at the user end [15], and we name it feedback compression-based imperfect CSI (FC-ICSI). Due to the limitation of feedback bandwidth or the requirement of feedback delays, users usually adopt as few feedback bits as possible to represent the feedback CSI; specific schemes include quantization and lossy compression [17]. Usually, the detected CSI would be quantized and compressed at the user end before it is sent to the central unit. Therefore, a loss of CSI in the received signals occurs at the central unit.

 The other two types of potential imperfect CSI are FF-ICSI and FDD-ICSI for short. The FF-ICSI is caused by the fast variation of channels and the feedback delay [17], and we call it fast-fading channel based imperfect CSI (FF-ICSI). Due to the Doppler effect caused by the object mobility, channel fading will vary quite fast. Because of the feedback delay, the estimated channel states may be different from the true CSI at the time of transmission. Therefore, even though the channel CSI in other parts of the system is perfect, the estimated CSI cannot represent the true CSI at the time of transmission. The FDD-ICSI depends on the duplexing scheme adopted in a system. If a system uses the frequency division duplexing (FDD) scheme, the forward channel and the feedback channel adopt different frequency bands [18]. In this case, the estimated CSI cannot represent the CSI of channels for data transmission, since the former is the CSI in forward directions, that is, from the central unit to users, and the latter is the CSI in feedback directions, that is, from users to the central unit. We call this type of imperfect CSI as FDD-based imperfect CSI (FDD-ICSI). In a WLAN in-hospital environments, the speed of patients is quite low, and the Doppler effect can be ignored [19]. Therefore, the fourth type of imperfect CSI, FF-ICSI, can be ignored in our analysis. As for the fifth type of imperfect CSI, FDD-ICSI, we can also ignore it, since a WLAN usually employs time division duplexing (TDD) instead of FDD [20].

### 2.3. Channel Models in Hospital Environments

 Generally, the characteristics of a wireless channel are mainly determined by the communication environment as well as the communication technology. In our case, the communication environment refers to the hospital and mainly depends on the hospital's building materials. The building materials in a hospital usually have unique characteristics, such as being electromagnetic interference resistant, weather resistant, fire proofing, temperature adaptable, and environment friendly [21]. Due to the unique characteristics of medical environment, the channel models widely used in general environment are not applicable in medical environment [19]. Additionally, even in the hospital environment, channel characteristics may also be different when a communication is at different wireless bands, which correspond to different attenuation of communication signals. We focus on using the IEEE 802.11n technology for communication within a hospital, and this technology employs the wireless bands around 2.4 GHz. Thus, the channel models at other wireless bands, such as those employed by the Oulu university hospital for ultra-wideband applications at bands from 3.1 GHz to 10.6 GHz [22], are not applicable in our case.

To investigate the unique channel characteristics around 2.4 GHz within a hospital, Huang et al. in [19] studied the channel models in three LOS and one NLOS case by taking channel measurements in the Kempenhaeghe Hospital, Heeze, the Netherlands. These three LOS cases include the transmission across the room (AR), the transmission along the front board of the bed (AB), and the transmission along the bedside (AS) [19], while the NLOS case refers to the transmission through the bed. By matching the measurements and widely used channel models, Huang et al. in [19] conclude that the channel fading in all LOS and NLOS cases can be modeled as Nakagami distributions with particular parameters, and these parameters for various cases are shown in Table 2. The Nakagami distribution of channel fading *A* can be expressed as

(1) $$f\left(A\right)=\frac{2m^{m}A^{2m-1}}{\sigma^{m}\Gamma \left(m\right)}e^{-mA^{2}/\sigma },$$

where Γ(·) is a Gamma function and *σ* and *m* are two determinant parameters of a Nakagami distribution. 

### 2.4. Related Work on the Prediction of Channel State Information

 To improve the efficiency of bandwidth allocation, researchers have proposed some algorithms to reduce the negative effects of each type of imperfect CSI. In this paper, we only focus on the algorithms to reduce the effects of imperfect CSI caused by the failure of CSI feedback. Due to the failure of CSI feedback at one transmission, the same CSI has to be retransmitted, and this retransmission would lead to a delay of CSI feedback. Once the delay is longer than one time slot, the central unit for bandwidth allocation cannot access the CSI of one particular user and has to estimate the CSI of this user. Therefore, an efficient algorithm to estimate CSI is necessary to reduce the effects caused by the failure of CSI feedback. Mielczarek and Krzymień in [3] propose a channel-prediction-based algorithm to estimate the current CSI in a centralized network. In this network, when the central unit cannot gather the CSI from a user, it would predict the current CSI by previous CSI, since the channel states are temporally correlated. The assumption in [3] is that the distribution of channel states is constant, but this assumption may not hold in reality, since the distribution of channel states is usually time variant. To predict time-variant channel state information, Mielczarek and Krzymień in [4] propose a dynamic channel prediction model, and the parameters of this model are updated in each time slot. This dynamic channel prediction model can attain the current CSI by extrapolating from previous CSI. The researchers in [5] and [6] also employ channel prediction to reduce the effects of feedback delay in the scenario of adaptive transmission and adaptive antenna selection.

 The relevant literature [3–6] assumes that the central unit for bandwidth allocation can gather the exact CSI at previous time slots to predict the CSI at current time slot. However, the central unit may not gather the exact previous CSI since the delay of CSI feedback may be several time slots. Thus, these algorithms proposed in the abovementioned literature cannot be applied into a practical scenario. Additionally, the literature [3–6] has not taken the scenario of healthcare monitoring into account, and the channel models employed in the literature cannot be applied for healthcare monitoring since different communication environments would lead to different channel characteristics (detailed in Section 2.3). The models in [3–6] are established in the scenario of offices or laboratories, in which communication channels are different from those in a hospital [19]. Given these reasons, we attempt to propose a channel-prediction-based algorithm to reduce the negative effects of imperfect CSI on bandwidth allocation within an in-hospital monitoring system.

## 3. Bandwidth Allocation Schemes without Channel Prediction

In this section, we first discuss the scheme to transmit medical data, which is the foundation of bandwidth allocation.

### 3.1. A Scheme to Transmit Medical Data

 As discussed in Section 2.1, we classify the traffic in the WLAN into three categories: real-time messages, real-time video conferences, and medical data. Thus, the traditional scheme for bandwidth allocation is to allocate all the subcarriers of WLAN among three parts [23]: a message part, an application part, and a data part. The message part is for the transmission of messages; the application part is for video conferences; the data part is for the transmission of medical data. However, the traditional scheme would lead to the underutilization of the application part, since video conferences do not occur all the time. The underutilization of bandwidth would further cause a smaller capacity of patients supported by the WLAN for in-hospital healthcare monitoring. The capacity of patients supported in a hospital is one of the most concerning problems in developing countries, in which the healthcare resources are usually insufficient for a large national population.

**Figure 3:**
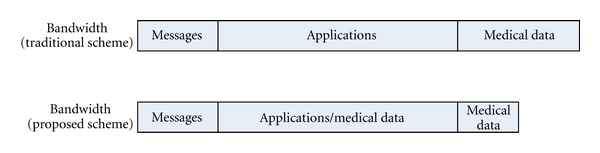
Proposed scheme of bandwidth allocation [24].

To enhance the capacity of patients supported by the WLAN in hospitals, we propose a novel scheme of data transmission in [24]. In this scheme, the application part is used to transmit medical data when not all the subcarriers allocated for video conference are busy. Therefore, we can store medical data in patient devices when all subcarriers are busy, while freeing the memory of devices by transmitting the stored medical data to the doctor's office when some subcarriers in the application part are idle. If the memory size of a patient device is unlimited and the required delay for data transmission is also unlimited, then we do not necessarily employ extra subcarriers to transmit medical data. However, in reality, the memory size of a patient device is limited and the required delay is also limited. Therefore, some extra subcarriers specially for the transmission of medical data are required even with the proposed scheme for data transmission, and we name these extra subcarriers as special subcarriers. The traditional scheme and the proposed scheme for data transmission are shown in Figure 3. In comparison with the traditional scheme, the proposed scheme can enhance the capacity of patients [24]. Therefore, the following discussion is building on the proposed scheme for data transmission.

### 3.2. Optimal Subcarrier Allocation in the Case of Perfect CSI

 Medical data have different priorities according to patient status, and the medical data with a higher priority have a more demanding requirement of transmission delay. Usually, the patient status can be classified into “high-degree (H),” “low-degree (L),” and “normal (N),” which represent the emergency degree of patient status [25]. Building on the proposed scheme of transmitting medical data in Section 3.1, we discuss the method to maximize the capacity of patients supported by the WLAN, given the maximal potential number of subcarriers supported by the WLAN.

Let *N*_*u*_ be the number of patients, *N*_*T*_ be the total number of time slots during monitoring, *S* ^(*k*)^ the number of special subcarriers in the *k*th (*k* = 1,2,…, *N*_*T*_) time slot, *M*_*i*_^(*k*)^ [bits] the amount of data in the memory of the *i*th patient device in the *k*th time slot, *B*_*i*_^(*k*)^ [Hz] the bandwidth allocated to the *i*th device in the *k*th time slot, *η*_*i*_^(*k*)^ [bps/Hz] the bandwidth efficiency of the *i*th device in the *k*th time slot, *a*_*i*_^(*k*)^ [bps] the data arrival rate of the *i*th device in the *k*th time slot, *M*_*i*_^max⁡^ [bits] the memory size of the *i*th device, *B*_*a*_^(*k*)^ [Hz] the bandwidth for applications in the *k*th time slot, *T*_*c*_ [s] the duration of one time slot, *B*_total_ [Hz] the total amount of bandwidth, Δ*B* [Hz] the bandwidth of one subcarrier, Δ*T*_1_ [s], Δ*T*_2_ [s], and Δ*T*_3_ [s] the tolerable delay for data transmission as the patient status is “H,” “L,” and “N,” respectively. For simplicity, we assume Δ*T*_3_ = *∞*; that is, the transmission of data corresponding to status “N” has no delay requirement. Then, the problem of maximizing the patient capacity can be modeled as a dynamic programming problem, and this dynamic programming problem in the *k*th (*k* = 1,2,…, *N*_*T*_) time slot can be denoted as

(2) $$\begin{array}{cc} \underset{B_{i}^{\left(k\right)}}{\mathrm{Max}} & \;N_{u}\\ \text{s}.\text{t}. & \;M_{i}^{\left(k\right)}=\mathrm{Max}\left\{\left(a_{i}^{\left(k\right)}-\eta_{i}^{\left(k\right)}B_{i}^{\left(k\right)}\right)T_{c}+M_{i}^{\left(k-1\right)},0\right\},\\ & \;M_{i}^{\left(k\right)}\leq M_{i}^{\max},\\ & \;\overset{N_{u}}{\underset{i=1}{\sum }}B_{i}^{\left(k\right)}+B_{a}^{\left(k\right)}=S^{\left(k\right)}\Delta B+\overset{N_{u}}{\underset{i=1}{\sum }}B_{i}^{\left(k-1\right)},\\ & \;\overset{N_{u}}{\underset{i=1}{\sum }}B_{i}^{\left(k\right)}+B_{a}^{\left(k\right)}\leq B_{\text{total}},\\ & \;a_{i}^{\left(k\right)}T_{c}\leq \eta_{i}^{\left(k\right)}B_{i}^{\left(k\right)}\Delta T_{1}\;\left(i\in H\right),\\ & \;a_{i}^{\left(k\right)}T_{c}\leq \eta_{i}^{\left(k\right)}B_{i}^{\left(k\right)}\Delta T_{2}\;\left(i\in L\right). \end{array}$$

As shown in (2), the objective of this dynamic programming is to find the maximal *N*_*u*_. In (2), the first constraint represents that the amount of data in the memory equals that in the last time slot plus the data accumulated in this time slot. Of course, the amount of data in the memory should be nonnegative. The second constraint ensures that the amount of data in the memory should be less than the memory size. The third constraint represents that the total bandwidth consists of the bandwidth employed for applications (video conferences) and that employed for transmitting medical data. Additionally, the total bandwidth in the *k*th time slot is equal to the total bandwidth in the (*k* − 1)th time slot plus the bandwidth of special subcarriers in the *k*th time slot. The fourth constraint ensures that the total bandwidth utilized in the WLAN should be less than the given bandwidth of a WLAN. The fifth and the sixth constraints ensure that the data in state “H” and “L” should satisfy delay requirements.

Equation (2) is a nondeterministic polynomial time (NP) problem. It is difficult to directly calculate the capacity of patients supported by the WLAN; therefore, we transfer the problem of maximizing the capacity of patients into the problem of minimizing the number of required subcarriers given the number of patients. Mathematically, the dynamic programming problem in the *k*th (*k* = 1,2,…, *N*_*T*_) time slot can be denoted as

(3) $$\begin{array}{cc} \underset{B_{i}^{\left(k\right)}}{\mathrm{Min}} & \;S^{\left(k\right)}\\ \text{s}.\text{t}.\; & \;M_{i}^{\left(k\right)}=\mathrm{Max}\left\{\left(a_{i}^{\left(k\right)}-\eta_{i}^{\left(k\right)}B_{i}^{\left(k\right)}\right)T_{c}+M_{i}^{\left(k-1\right)},0\right\},\\ & \;M_{i}^{\left(k\right)}\leq M_{i}^{\max},\\ & \;\overset{N_{u}}{\underset{i=1}{\sum }}B_{i}^{\left(k\right)}+B_{a}^{\left(k\right)}=S^{\left(k\right)}\Delta B+B^{\left(k-1\right)},\\ & \;\overset{N_{u}}{\underset{i=1}{\sum }}B_{i}^{\left(k\right)}+B_{a}^{\left(k\right)}\leq B_{\text{total}},\\ & \;a_{i}^{\left(k\right)}T_{c}\leq \eta_{i}^{\left(k\right)}B_{i}^{\left(k\right)}\Delta T_{1}\;\left(i\in H\right),\\ & \;a_{i}^{\left(k\right)}T_{c}\leq \eta_{i}^{\left(k\right)}B_{i}^{\left(k\right)}\Delta T_{2}\;\left(i\in L\right). \end{array}$$

In (3), the objective is to minimize *S* ^(*k*)^ in each slot, which would lead to the minimization of ∑_*k*=1_^*N*_*T*_^ *S* ^(*k*)^. In each time slot, the available solutions to this dynamic programming may not be unique. Therefore, we select the one to maximize the overall data rate, that is, to maximize ∑_*i*=1_^*N*_*u*_^ *η*_*i*_^(*k*)^ *B*_*i*_^(*k*)^. For a given *N*_*u*_, (3) is a linear programming problem; it can be solved by any standard method. We have used the MATLAB toolbox for integer linear programming problems.

For each given *N*_*u*_, we can calculate the minimal number of subcarriers for data transmission. Then, we increase *N*_*u*_, and the number of required subcarriers also increase; the maximal number of patients supported by the WLAN can be obtained as the number of required subcarriers increase to *B*_total_/Δ*B*.

### 3.3. Optimal Subcarrier Allocation in the Case of Imperfect CSI

 We have discussed bandwidth allocation in the scenario of perfect CSI in Section 3.2. In reality, the CSI is usually not perfect due to channel estimation errors, feedback errors, and feedback delays; these types of imperfect CSI would lead to the deterioration of system performance.

#### 3.3.1. Effect of Type I Imperfect CSI

 The first type of imperfect CSI is caused by the error of channel estimation; the errors are usually modeled as complex circular Gaussian random variables, and the accuracy of this model is shown in [26]. Based on the model of channel estimation errors, an estimated bandwidth efficiency ${\bar{\eta }}_{i}^{\left(k\right)}$ in the MIMO-OFDM-based WLAN can be expressed as (4) [26]. In (4), [*x*]_*i*_^(*k*)^ represents that the value is for the *i*th user in the *k*th time slot; *m* = min⁡{*n*_*T*_, *n*_*R*_} and *n* = max⁡{*n*_*T*_, *n*_*R*_}, given that *n*_*T*_ and *n*_*R*_ are the number of transit antennas and receive antennas, respectively; *a*_*p*,*l*_ is a coefficient, and the detailed calculation process is presented in [26]; *r* is the average signal-to-noise ratio of the transmitted data; *σ*_*e*_^2^ is the variance of channel estimation errors; *E*_*l*_(*x*) = ∫_1_^*∞*^ *e* ^−*xt*^ *t* ^−*l*^ *dt* is named the *l*-order exponential integral function of *x*; *f*(*σ*_*e*_^2^; *r*) = *r*/((1 + *σ*_*e*_^2^)(1 + *σ*_*e*_^2^(1 + *r*))).

It is easy to show that exp⁡(*x*)*E*_*l*+2−*j*_(*x*) decreases with *x* and 1/*f*(*σ*_*e*_^2^; *r*) increases with *σ*_*e*_^2^ for any *j*, *l*, *p*; according to the following, ${\bar{\eta }}_{i}^{\left(k\right)}$ decreases with *σ*_*e*_^2^. Therefore, ${\bar{\eta }}_{i}^{\left(k\right)}\leq \eta_{i}^{\left(k\right)}$; *η*_*i*_^(*k*)^ is the exact bandwidth efficiency, that is, the bandwidth efficiency as *σ*_*e*_^2^ = 0.

(4) $$\begin{array}{cc} {\overset{̅}{\eta }}_{i}^{\left(k\right)} & =[\overset{m}{\underset{p=1}{\sum }}\exp\left\{\frac{p}{f\left(\sigma_{e}^{2};r\right)}\right\}\\ & \;{\overset{(n+m-2p)p}{\underset{l=n-m}{\sum }}a_{p,l}\overset{l+1}{\underset{j=1}{\sum }}E_{l+2-j}\left(\frac{p}{f\left(\sigma_{e}^{2};r\right)}\right)]}_{i}^{\left(k\right)}. \end{array}$$

#### 3.3.2. Effect of Type II Imperfect CSI

 Type II imperfect CSI is caused by the failure of CSI feedback, and the probability of failure is denoted as *p*_*e*_. We employ the ARQ scheme with *M* retransmission at most. Then, the ${\bar{\eta }}_{i}^{\left(k\right)}$ can be expressed as

(5) $$\begin{array}{c} {\bar{\eta }}_{i}^{\left(k\right)}=\{\begin{array}{cc} \eta_{i}^{\left(k\right)}, & \text{successful}\;\text{CSI}\;\text{feedback},\\ \eta_{0}, & \text{unsuccessful}\;\text{CSI}\;\text{feedback}, \end{array} \end{array}$$

where *η*_0_ is a predefined bandwidth efficiency as the CSI transmission failed in feedback. The probability of unsuccessful CSI feedback is (*p*_*e*_)^*M*^, and the probability of successful CSI feedback is 1 − (*p*_*e*_)^*M*^. Therefore, from the perspective of probability, the ${\bar{\eta }}_{i}^{\left(k\right)}$ can also be expressed as

(6) $$\begin{array}{c} {\bar{\eta }}_{i}^{\left(k\right)}=\{\begin{array}{cc} \eta_{i}^{\left(k\right)}, & \text{with}\;\text{a}\;\text{probability}\;\text{of}\;1-{\left(p_{e}\right)}^{M},\\ \eta_{0}, & \text{with}\;\text{a}\;\text{probability}\;\text{of}\;{\left(p_{e}\right)}^{M}. \end{array} \end{array}$$

#### 3.3.3. Effect of Type III Imperfect CSI

 Type III imperfect CSI is caused by the lossy data compression; the estimated bandwidth efficiency in the feedback is not the exact bandwidth efficiency but represented by a smaller number of information bits. Mathematically, the ${\bar{\eta }}_{i}^{\left(k\right)}$ can be represented as

(7) $$\begin{array}{c} {\bar{\eta }}_{i}^{\left(k\right)}={\left[\eta_{i}^{\left(k\right)}\right]}_{l}, \end{array}$$

where *η*_*i*_^(*k*)^ is the exact bandwidth efficiency; [*x*]_*l*_ means that the value of *x* is represented by *l* bits of information. For instance, if we assume that *η*_*i*_^(*k*)^ ∈ [0,1], then we divide the section [0,1] into 2^*l*^ parts and estimate the value of *x* as *m*/2^*l*^  (*m* = 0 ⋯ 2^*l*^ − 1), when *x* falls into the *m*th part.

Building on (4) and (5), the overall ${\bar{\eta }}_{i}^{\left(k\right)}$ can be expressed as (8) (on page 7). Here *η*_0_ is a predefined bandwidth efficiency as the channel estimation failed. The probability of unsuccessful channel estimation is (*p*_*e*_)^*M*^, and the probability of successful channel estimation is 1 − (*p*_*e*_)^*M*^. Therefore, from the perspective of probability, the ${\bar{\eta }}_{i}^{\left(k\right)}$ can also be expressed as (9) (on page 7):

(8) $$\begin{array}{cc} {\bar{\eta }}_{i}^{\left(k\right)} & =\{\begin{array}{cc} {\left[{\left[\overset{m}{\underset{p=1}{\sum }}\exp\left\{\frac{p}{f\left(\sigma_{e}^{2};r\right)}\right\}\overset{(n+m-2p)p}{\underset{l=n-m}{\sum }}a_{p,l}\overset{l+1}{\underset{j=1}{\sum }}E_{l+2-j}\left(\frac{p}{f\left(\sigma_{e}^{2};r\right)}\right)\right]}_{l}\right]}_{i}^{\left(k\right)} & \text{success}\\ \eta_{0}\; & \text{failure}, \end{array} \end{array}$$

(9) $$\begin{array}{cc} {\bar{\eta }}_{i}^{\left(k\right)} & =\{\begin{array}{cc} {\left[{\left[\overset{m}{\underset{p=1}{\sum }}\exp\left\{\frac{p}{f\left(\sigma_{e}^{2};r\right)}\right\}\overset{(n+m-2p)p}{\underset{l=n-m}{\sum }}a_{p,l}\overset{l+1}{\underset{j=1}{\sum }}E_{l+2-j}\left(\frac{p}{f\left(\sigma_{e}^{2};r\right)}\right)\right]}_{l}\right]}_{i}^{\left(k\right)} & \text{with}\;1-{\left(p_{e}\right)}^{M}\\ \eta_{0} & \text{with}\;{\left(p_{e}\right)}^{M}. \end{array} \end{array}$$

## 4. Bandwidth Allocation Schemes with Channel Prediction

 In Section 3.3, we discussed the effects of imperfect CSI on the performance of bandwidth allocation. In this section, we propose an algorithm to reduce the negative effects of type II imperfect CSI. To familiarize readers, we firstly discuss the channel prediction models in hospital environments as backgrounds. Then, we propose the specific algorithm to reduce the effects of type II imperfect CSI. Finally, we analyze the reliability of our proposed algorithm.

### 4.1. Channel Prediction Model in Hospital Environments

 Building on the Nakagami fading channels in (1), we further discuss the channel prediction model in hospital environments, that is, the model to predict CSI given channel characteristics. Specifically, as for a Nakagami channel model, the channel characteristics are determined by parameters *σ* and *m*.

Denote *α*_*t*−*τ*_ and *α*_*t*_ as the channel fades at the time of *t* − *τ* and *t*, respectively. Then, the joint probability density function (pdf) of *α*_*t*−*τ*_ and *α*_*t*_ can be expressed as [27]:

(10) $$\begin{array}{cc} f\left(\alpha_{t-\tau },\alpha_{t}\right) & =\frac{4{\left(\alpha_{t-\tau }\alpha_{t}\right)}^{m}}{\left(1-\rho \right)\Gamma \left(m\right)\rho^{(m-1)/2}}{\left(\frac{m}{\sigma }\right)}^{m+1}\\ & \;I_{m-1}\left(\frac{2m\sqrt{\rho }\alpha_{t-\tau }\alpha_{t}}{\left(1-\rho \right)\sigma }\right)\exp\left(-\frac{m\left(\alpha_{t-\tau }^{2}+\alpha_{t}^{2}\right)}{\left(1-\rho \right)\sigma }\right), \end{array}$$

where *I*_*m*−1_(·) is the (*m* − 1)th order modified Bessel function; Γ(·) is a Gamma function; *σ* and *m* are the Nakagami fading parameters shown in (5); *ρ* is the channel correlation, given that [28]

(11) $$\begin{array}{c} \rho \left(\tau \right)={\left(\frac{I_{0}\left(\sqrt{\kappa^{2}-{\left(2\pi f_{D}\tau \right)}^{2}+j4\pi \kappa f_{D}\tau \cos\mu }\right)}{I_{0}\left(\kappa \right)}\right)}^{2}, \end{array}$$

where *f*_*D*_ is the maximum Doppler frequency (in hertz), *μ* is the mean direction of the angle of arrival (AOA), and *κ* is the beamwidth parameter.

The channel states can be represented by *L* channel states; namely, *s*_*j*_(*j* = 1 … *L*) [29]; *s*_*j*_ is related to the threshold of Signal to Noise Ratio (SNR) *r*_*j*_^*t*^ and the corresponding threshold of bandwidth efficiency *η*_*j*_^*t*^. As the event of SNR ∈ [*r*_*j*−1_^*t*^, *r*_*j*_^*t*^) occurs, the corresponding channel state is *s*_*j*_(*j* = 1,…, *L*). Let *x*_*i*_ and *x*_*i*+1_ denote the SNR at the *i*th and (*i* + 1)th time slot, and let *η*_*i*_ and *η*_*i*+1_ denote the corresponding bandwidth efficiency at the *i*th and *i* + 1th time slot, respectively. Then, the given probability *p*_*jk*_ can be expressed as [28]

(12) $$\begin{array}{cc} p_{jk} & =P\left(\eta_{i+1}\in \left(\eta_{k-1}^{t},\eta_{k}^{t}\right)\;|\;\eta_{i}\in \left(\eta_{j-1}^{t},\eta_{j}^{t}\right)\right)\\ & =P\left(x_{i+1}\in \left(r_{k-1}^{t},r_{k}^{t}\right)\;|\;x_{i}\in \left(r_{j-1}^{t},r_{j}^{t}\right)\right)\\ & =\frac{P\left(x_{i+1}\in \left(r_{k-1}^{t},r_{k}^{t}\right),x_{i}\in \left(r_{j-1}^{t},r_{j}^{t}\right)\right)}{P\left(x_{i}\in \left(r_{j-1}^{t},r_{j}^{t}\right)\right)}\\ & =\frac{\int_{r_{k-1}^{t}}^{r_{k}^{t}}\int_{r_{j-1}^{t}}^{r_{j}^{t}}f\left(\alpha_{t-\tau }\alpha_{t}\right)d\alpha_{t-\tau }d\alpha_{t}}{\int_{0}^{\infty }\int_{r_{j-1}^{t}}^{r_{j}^{t}}f\left(\alpha_{t-\tau }\alpha_{t}\right)d\alpha_{t-\tau }d\alpha_{t}}. \end{array}$$

Submitting (10) into (12), we can calculate the transition probability of channel states and predict the future channel states given the past states. Equation (12) shows the one-step transition between any two particular channel states, and all the possible transition forms a matrix, which is denoted as a one-step transition matrix. Mathematically, given *L* channel states in total, the one-step transition matrix can be expressed as

(13) $$P=\left[\begin{array}{ccc} p_{11} & \cdots & p_{L1}\\ \vdots & \ddots & \vdots \\ p_{1L} & \cdots & p_{LL} \end{array}\right].$$

The Nakagami fading channel can be viewed as a Markov chain [30], since a higher-order Markov model can well represent the Nakagami channels [31]. Due to the properties of Markov chains, the M-step transition matrix can be calculated as *P* ^*M*^. In reality, the one-step transition matrix in (13) can be statistically estimated by averaging the observations over long periods of time.

### 4.2. Channel-Prediction-Based Algorithm to Reduce the Effects of Type II Imperfect CSI

 We assume that the distribution of channel characteristics is constant within *N* time slots, and this assumption is reasonable, because the channel characteristics in the scenario of in-hospital monitoring are slow-fading due to the low-speed mobility of patients or doctors. Building on the channel prediction model in hospital environments discussed in Section 4.2, the channel prediction based algorithm to reduce the effects caused by feedback delay can be described as Algorithm 1.

In Algorithm 1, the variation of channel characteristics has been taken into account, and the parameters of channel models are updated every *N* time slots in step (1). Additionally, the algorithm also considers the case that not all the CSI of previous time slots is gathered by the central unit; in this case, the latest CSI is employed to estimate the CSI of the current time slot in step (3) and step (4). One extreme case is that none of CSI in previous *N* time slots is gathered by the central unit, and, in this case, the central unit can only estimate the CSI as a predefined *η*_0_.

### 4.3. Optimal Subcarrier Allocation with Channel Prediction

 In our proposed algorithm, the overall ${\bar{\eta }}_{i}^{\left(k\right)}$ can be expressed as (14). In (14), *η*_(out)*i*_^(*k*)^ is the estimated CSI by Algorithm 1 if the transmission of current CSI failed. The probability of unsuccessful channel estimation is (*p*_*e*_)^*M*^, and the probability of successful channel estimation is 1 − (*p*_*e*_)^*M*^. Therefore, from the perspective of probability, ${\bar{\eta }}_{i}^{\left(k\right)}$ can also be expressed as (15):

(14) $$\begin{array}{cc} {\bar{\eta }}_{i}^{\left(k\right)} & =\{\begin{array}{cc} {\left[{\left[\overset{m}{\underset{p=1}{\sum }}\exp\left\{\frac{p}{f\left(\sigma_{e}^{2};r\right)}\right\}\overset{(n+m-2p)p}{\underset{l=n-m}{\sum }}a_{p,l}\overset{l+1}{\underset{j=1}{\sum }}E_{l+2-j}\left(\frac{p}{f\left(\sigma_{e}^{2};r\right)}\right)\right]}_{l}\right]}_{i}^{\left(k\right)} & \text{success}\\ \eta_{\left(\text{out}\right)i}^{\left(k\right)} & \text{failure}, \end{array} \end{array}$$

(15) $$\begin{array}{cc} {\bar{\eta }}_{i}^{\left(k\right)} & =\{\begin{array}{cc} {\left[{\left[\overset{m}{\underset{p=1}{\sum }}\exp\left\{\frac{p}{f\left(\sigma_{e}^{2};r\right)}\right\}\overset{(n+m-2p)p}{\underset{l=n-m}{\sum }}a_{p,l}\overset{l+1}{\underset{j=1}{\sum }}E_{l+2-j}\left(\frac{p}{f\left(\sigma_{e}^{2};r\right)}\right)\right]}_{l}\right]}_{i}^{\left(k\right)} & \text{with}\;1-{\left(p_{e}\right)}^{M}\\ \eta_{\left(\text{out}\right)i}^{\left(k\right)} & \text{with}\;{\left(p_{e}\right)}^{M}. \end{array} \end{array}$$

## 5. Analyzing the Reliability of Proposed Algorithm

 In this channel-prediction-based algorithm, we employ the maximum a posteriori (MAP) prediction; that is, *η*_(out)*i*_^(*k*)^ = arg max⁡_*j*={1,2,…,*L*}_ *p*_*jl*_, given the previous CSI in state *l* and user *i*. Due to the limitation of MAP, this algorithm may lead to an error at the prediction of CSI with a probability of (1 − *p*_*jl*_) even when the previous CSI is assumed perfect. Additionally, the CSI at previous time slots gathered by the central unit may be imperfect due to the type I and type III imperfect CSI, and the imperfect CSI would also lead to an error at the prediction of CSI even as *p*_*jl*_ = 1. In reality, the imperfect previous CSI and the limitation of MAP scheme would lead to the errors of channel prediction in joint. In the following, we analyze the joint effects on channel prediction.

Firstly, we assume that *η* ^(*k*−*n*)^, the CSI at the (*k* − *n*)th time slot, is perfect. When we employ the perfect previous CSI to predict the current CSI, the errors of prediction are caused by the limitation of MAP scheme. Let *η* ^(*k*−*n*)^ = *l* and *η*_(out)*i*_^(*k*)^ = arg max⁡_*j*={1,2,…,*L*}_ *p*_*jl*_. Given *η*_(out)*i*_^(*k*)^ = *t*, the mean square errors (MSE) between *η*_(out)*i*_^(*k*)^ and *η*_*i*_^(*k*)^, we say MSE_1_, can be expressed as

(16) $$\begin{array}{cc} \text{MS}{\text{E}}_{1} & =E\left\{{\left(\eta_{\left(\text{out}\right)i}^{k}-\eta_{i}^{k}\right)}^{2}|t=\underset{j=\left\{1,2,\ldots ,L\right\}}{\arg\;\max}p_{jl}\right\}\\ & =E\left\{{\left(\eta_{\left(\text{out}\right)i}^{k}-{\left[\eta_{i}^{k}\right]}_{Q}+{\left[\eta_{i}^{k}\right]}_{Q}-\eta_{i}^{k}\right)}^{2}|t=\underset{j=\left\{1,2,\ldots ,L\right\}}{\arg\;\max}p_{jl}\right\}\\ & =E\left\{{\left(\eta_{\left(\text{out}\right)i}^{k}-{\left[\eta_{i}^{k}\right]}_{Q}\right)}^{2}|t=\underset{j=\left\{1,2,\ldots ,L\right\}}{\arg\;\max}p_{jl}\right\}\\ & \;+E\left\{{\left({\left[\eta_{i}^{k}\right]}_{Q}-\eta_{i}^{k}\right)}^{2}|t=\underset{j=\left\{1,2,\ldots ,L\right\}}{\arg\;\max}p_{jl}\right\}\\ & \;+2E\left\{\eta_{\left(\text{out}\right)i}^{k}-{\left[\eta_{i}^{k}\right]}_{Q}|t=\underset{j=\left\{1,2,\ldots ,L\right\}}{\arg\;\max}p_{jl}\right\}\\ & \;\times E\left\{{\left[\eta_{i}^{k}\right]}_{Q}-\eta_{i}^{k}|t=\underset{j=\left\{1,2,\ldots ,L\right\}}{\arg\;\max}p_{jl}\right\}, \end{array}$$

where *E*{*x*} represents the mean of *x* and [*η*_*i*_^*k*^]_*Q*_ represents the quantization of *η*_*i*_^*k*^, the perfect CSI at the *k*th time slot, by flooring to the nearest quantized state. In (16), we also assume that the quantization errors at the *k*th time slot are independent of the errors due to the limitation of MAP scheme.

In view of (16), the MSE_1_ is composed of three terms. In the following, we calculate these terms, respectively. The first term can be expressed as

(17) $$\begin{array}{cc} & E\left\{{\left(\eta_{\left(\text{out}\right)i}^{k}-{\left[\eta_{i}^{k}\right]}_{Q}\right)}^{2}|t=\underset{j=\left\{1,2,\ldots ,L\right\}}{\arg\;\max}p_{jl}\right\}\\ & =\overset{L}{\underset{j=1}{\sum }}p_{jl}{\left(t-j\right)}^{2}\Delta^{2}, \end{array}$$

where Δ is the step size of uniform quantization.

The second term of MSE_1_ refers to the variance of quantization errors, and it can be expressed as

(18) $$E\left\{{\left({\left[\eta_{i}^{k}\right]}_{Q}-\eta_{i}^{k}\right)}^{2}|t=\underset{j=\left\{1,2,\ldots ,L\right\}}{\arg\;\max}p_{jl}\right\}=\frac{\Delta^{2}}{12}.$$

The third term of MSE_1_ is the joint term, and it can be expressed as

(19) $$\begin{array}{cc} & 2E\left\{\eta_{\left(\text{out}\right)i}^{k}-{\left[\eta_{i}^{k}\right]}_{Q}|t=\underset{j=\left\{1,2,\ldots ,L\right\}}{\arg\;\max}p_{jl}\right\}\\ & \;\times E\left\{{\left[\eta_{i}^{k}\right]}_{Q}-\eta_{i}^{k}|t=\underset{j=\left\{1,2,\ldots ,L\right\}}{\arg\;\max}p_{jl}\right\}\\ & =2\overset{L}{\underset{j=1}{\sum }}p_{jl}\left(j-t\right)\Delta \frac{\Delta }{2}=\overset{L}{\underset{j=1}{\sum }}p_{jl}\left(j-t\right)\Delta^{2}. \end{array}$$

In addition, we calculate *M*_1_, the mean of *η*_(out)*i*_^*k*^ − *η*_*i*_^*k*^, as

(20) $$\begin{array}{cc} M_{1} & =E\left\{\eta_{\left(\text{out}\right)i}^{k}-\eta_{i}^{k}|t=\underset{j=\left\{1,2,\ldots ,L\right\}}{\arg\;\max}p_{jl}\right\}\\ & =\overset{L}{\underset{j=1}{\sum }}p_{jl}\left(j-t\right)\Delta +\frac{\Delta }{2}. \end{array}$$

Secondly, we consider the case that ${\bar{\eta }}^{\left(k-n\right)}$, the CSI at the (*k* − *n*)th time slot, is imperfect. Then, the errors of prediction are caused by both the limitation of MAP and imperfect CSI at previous time slots. Let ${\bar{\eta }}^{\left(k-n\right)}=\bar{l}$. Due to type I imperfect CSI, the ${\bar{\eta }}^{\left(k-n\right)}$ may correspond to a different quantized state with the perfect CSI *η* ^(*k*−*n*)^ = *l*; that is, $\bar{l}\neq l$. From the perspective of MAP prediction, due to the imperfect CSI at the (*k* − *n*)th time slot, *η*_(out)*i*_^(*k*)^ may be incorrectly predicted based on the $\bar{l}$th column of *P*, instead of the *l*th column of *P*. Drawing from (16) and (20), the mean square errors (MSEs) between *η*_(out)*i*_^(*k*)^ and *η*_*i*_^(*k*)^, the perfect CSI at the *k*th time slot, we say MSE_2_, can be expressed as

(21) $$\begin{array}{cc} \text{MS}{\text{E}}_{2} & =E\left\{{\left(\eta_{\left(\text{out}\right)i}^{k}-\eta_{i}^{k}\right)}^{2}|t=\underset{j=\left\{1,2,\ldots ,L\right\}}{\arg\;\max}p_{jl}\right\}\\ & =\text{MS}{\text{E}}_{1}+E\left\{{\left(\eta_{\left(\text{out}\right)i}^{k}-{\underline{\eta }}_{\left(\text{out}\right)i}^{k}\right)}^{2}|t=\underset{j=\left\{1,2,\ldots ,L\right\}}{\arg\;\max}p_{jl}\right\}\\ & \;+2M_{1}E\left\{\eta_{\left(\text{out}\right)i}^{k}-{\underline{\eta }}_{\left(\text{out}\right)i}^{k}|t=\underset{j=\left\{1,2,\ldots ,L\right\}}{\arg\;\max}p_{jl}\right\}, \end{array}$$

where ${\underline{\eta }}_{(\text{out})i}^{k}$ represents the CSI at time slot *k* when the CSI at time slot *k* − *n* is perfect. In (21), we assume that the errors caused by the imperfect CSI at time slot *k* − *n* are independent from the errors due to the limitation of MAP scheme.

In view of (21), the MSE_2_ is composed of three terms. In the following, we calculate these terms, respectively. The first term is MSE_1_, and it is expressed as (16). The second term of MSE_2_ can be expressed as

(22) $$\begin{array}{c} E\left\{{\left(\eta_{\left(\text{out}\right)i}^{k}-{\underline{\eta }}_{\left(\text{out}\right)i}^{k}\right)}^{2}|t=\underset{j=\left\{1,2,\ldots ,L\right\}}{\arg\;\max}p_{jl}\right\}\\ =\sum_{\bar{t}}{\left(\bar{t}-t\right)}^{2}p\left(\bar{t}=\underset{j=\left\{1,2,\ldots ,L\right\}}{\arg\;\max}p_{j\bar{l}}\right)\Delta^{2}. \end{array}$$

The third term of MSE_2_ can be expressed as

(23) $$\begin{array}{c} 2M_{1}E\left\{\eta_{\left(\text{out}\right)i}^{k}-{\underline{\eta }}_{\left(\text{out}\right)i}^{k}|t=\underset{j=\left\{1,2,\ldots ,L\right\}}{\arg\;\max}p_{jl}\right\}\\ =2M_{1}\sum_{\bar{t}}\left(\bar{t}-t\right)p\left(\bar{t}=\underset{j=\left\{1,2,\ldots ,L\right\}}{\arg\;\max}p_{j\bar{l}}\right)\Delta , \end{array}$$

where *M*_1_ is shown as (20).

The prior probability *p*(*t* = arg max⁡_*j*={1,2,…,*L*}_ *p*_*jl*_) and $p(\bar{t}={\arg\;\max}_{j=\{1,2,\ldots ,L\}}p_{j\bar{l}})$ depend on the channel characteristics, shown in (12) and (13), respectively. Drawing from (21), we can deduce the MSE of bandwidth efficiency with the proposed prediction scheme as

(24) $$\begin{array}{c} \text{MSE}=\overset{L}{\underset{t=1}{\sum }}\text{MS}{\text{E}}_{2}\times p\left(t=\underset{j=\left\{1,2,\ldots ,L\right\}}{\arg\;\max}p_{jl}\right). \end{array}$$

Then, by dividing the exact bandwidth efficiency *η*_*i*_^*k*^, the relative standard error (RSE) with the prediction-based algorithm can be expressed as

(25) $$\begin{array}{c} \text{RSE}=\frac{\sqrt{\text{MSE}}}{\eta_{i}^{k}}, \end{array}$$

where MSE is shown in (24).

Similarly, we can also calculate the RSE of the algorithm that estimates the bandwidth efficiency as a predefined value *η*_0_. The RSE of the algorithm without channel prediction can be expressed as

(26) $$\begin{array}{c} \text{RS}{\text{E}}_{0}=\frac{\sqrt{E\left\{{\left(\eta_{0}-\eta_{i}^{k}\right)}^{2}\right\}}}{\eta_{i}^{k}}, \end{array}$$

where *η*_0_ is as (9).

## 6. Simulation and Discussion

 The parameters in the simulation are as follows: *N* = 10000; Δ*B* = 0.8 Mbps; *a*_*i*_^(*k*)^ = 800 kbps; *T*_*c*_ = 0.1 s; *B*_*a*_^(*k*)^/Δ*B* ~ *B*(*N*_*u*_, *p*), where *B*(·) represents a binomial distribution and *p* represents the probability of a subcarrier being occupied for video conferences. The parameter of the Nakagami distribution *m* is randomly selected among various parameters shown in Table 2. Additionally, we consider all three types of imperfect CSI in our simulation, since all of them would occur in reality.

**Figure 4:**
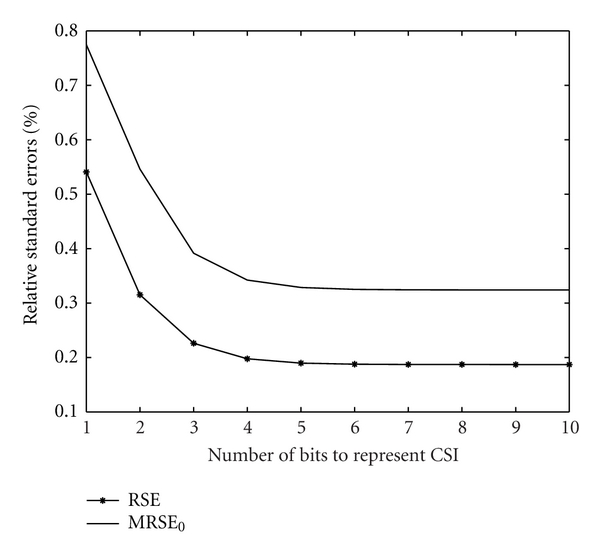
Relative standard errors of bandwidth efficiency.

Firstly, we discuss the reliability of the proposed algorithm by measuring the RSE of this algorithm, shown in (25). For comparison, we also take into account the RSE of the algorithm without channel prediction. As shown in (25), the RSE varies with the predefined channel efficiency, *η*_0_. We choose the minimum RSE among various *η*_0_; that is, MRSE_0_ = min⁡_*η*_0__ RSE_0_. In view of (25) and (26), the result of RSE is shown in Figure 4. As shown in Figure 4, the channel-prediction-based algorithm can reduce the RSE in comparison with the algorithm without channel prediction. The reasons for these results are as follows: the channels for in-hospital monitoring are slow fading, and the CSI between two time slots is temporally correlated. The channel-prediction-based algorithm employs the correlation between CSI at different time slots, while the algorithm without channel prediction has not utilized the channel correlation. Therefore, channel-prediction-based algorithm can attain a smaller RSE than the algorithm without channel prediction.

**Figure 5:**
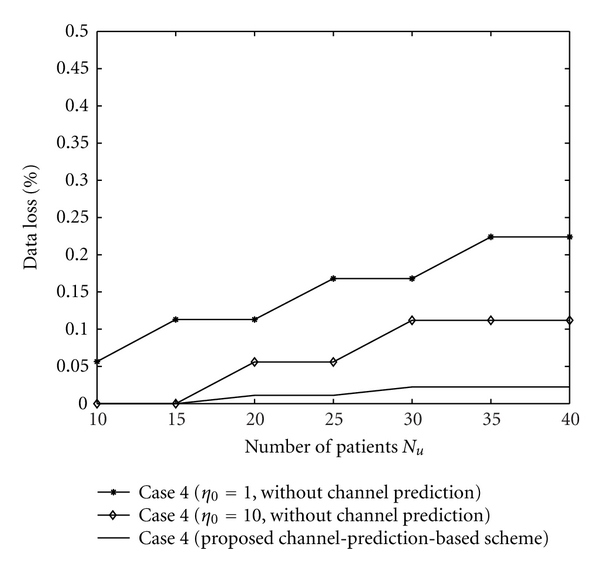
Data loss with the proposed algorithm and with the algorithm without channel prediction.

**Figure 6:**
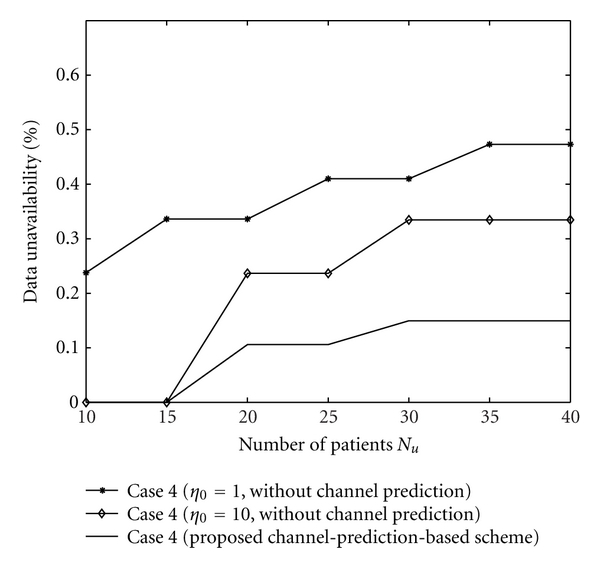
Data unavailability with the proposed algorithm and with the algorithm without channel prediction.

Secondly, we discuss the data loss and data unavailability caused by the errors of CSI estimation when the CSI at current time slot is unavailable. The data loss and data unavailability with the proposed algorithm and with the algorithm without channel prediction are shown in Figures 5 and 6. Figures 5 and 6 show that the channel-prediction-based algorithm proposed in this paper can reduce the data loss and data unavailability. The reasons are as follows: data loss and data unavailability occur when the estimated CSI is larger than the exact CSI. With the rise of the difference between estimated CSI and exact CSI, data loss and data unavailability would also increase. The channel-prediction-based algorithm can attain a smaller difference between the estimated CSI and the exact CSI than the algorithm with a predefined CSI (without channel prediction). Therefore, the proposed algorithm would attain lower data loss and data unavailability.

**Figure 7:**
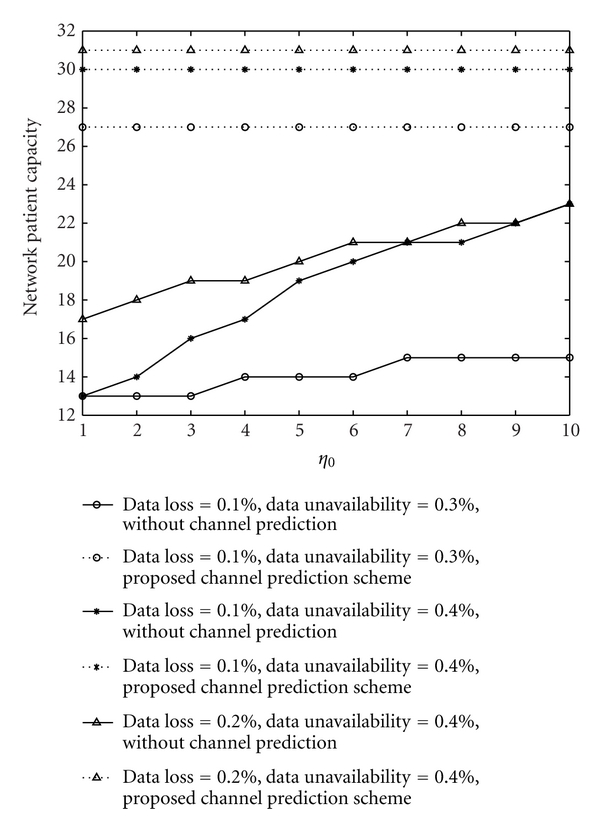
Network patient capacity with the proposed algorithm and that with the algorithm without channel prediction.

Thirdly, we discuss network patient capacity of the healthcare monitoring system. The network patient capacity refers to the maximal number of patients supported by the system, subject to limited wireless bandwidth, the requirements of data loss, and the requirements of data uncertainty. Given the fixed amount of wireless bandwidth, the network patient capacity depends on the requirements of data loss and data uncertainty. For various requirements of data loss and data uncertainty, the network patient capacity with the proposed algorithm and with the algorithm without channel prediction is shown in Figure 7. Figure 7 shows that the proposed algorithm can attain a larger network patient capacity than the algorithm without channel prediction. The reason is that when the algorithm without channel prediction attains the same network patient capacity as the proposed algorithm, the data loss and data uncertainty cannot be accepted for healthcare monitoring, because the former would lead to a higher data loss and data uncertainty than the latter.

## 7. Conclusions

 In this paper, we propose an algorithm that predicts CSI to reduce the negative effects of imperfect CSI caused by the failure of CSI feedback. In this algorithm, when the central unit for bandwidth allocation cannot access the current CSI, it would predict the current CSI by the CSI at previous time slots. To show the reliability of the proposed algorithm, we deduce the RSE between the estimated CSI and the exact CSI; then, the RSE with the proposed algorithm and that with an algorithm without channel prediction are compared. The results show that the proposed algorithm can attain a lower RSE than the algorithm without channel prediction. Then, to show the efficiency of the proposed algorithm on system performance, we compare the system performance with the proposed algorithm and that with the algorithm without channel prediction. The results show that the proposed algorithm can attain lower probability of data loss, lower probability of data unavailability, and larger network patient capacity.

 Regarding the resource allocation problem, we may consider using alternative algorithms to accelerate the solution to this optimization problem. Also we may consider using some alternative methods, such as the Kalman filter or stochastic robust control, to predict the channel state information. However, these issues are beyond the scope of this paper.

## Figures and Tables

**Algorithm 1:**
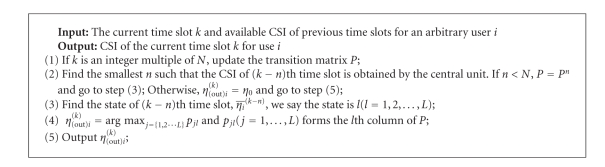
Channel-prediction-based algorithm.

**Table 1:** Information about ECG [7].

Number of leads	2–32
Samples per lead per second	200–500
Sample size (bit)	8, 16, 32

**Table 2:** Parameters of Nakagami channels (*m*) in various scenarios for in-hospital monitoring [19].

	Distance (*m*)		
	0.5	1	2
LOS/AR	2.8998	1.684	1.6116
LOS/AS	2.1665	1.6637	1.6356
LOS/AB	1	1	1
NLOS	1	1	1
